# Supplementary material for: Broadband random optoelectronic oscillator
Source: Nat Commun. 2020 Nov 12;11:5724. doi: 10.1038/s41467-020-19596-x (PMC7665046; doi:10.1038/s41467-020-19596-x)
Supplement: Supplementary file 1 — Supplementary Information [file 41467_2020_19596_MOESM1_ESM.pdf]

Supplementary Information for

Broadband Random Optoelectronic Oscillator

Broadband Random Optoelectronic Oscillator

Authors:

Zengting Ge[1,2], Tengfei Hao[1,2], José Capmany[3], Wei Li[1,2], Ninghua Zhu[1,2], Ming Li,[1,2*]

Affiliations:

[1] State Key Laboratory on Integrated Optoelectronics, Institute of Semiconductors, Chinese Academy of Sciences, Beijing 100083, China

[2] School of Electronic, Electrical and Communication Engineering, University of Chinese Academy of Sciences, Beijing 100049, China

[3] Photonics Research Labs, ITEAM Research Institute, Universitat Politecnica de Valencia, Camino de Vear s/n, 46022 Valencia, Spain

E-mail:

*e-mail: ml@semi.ac.cn

Supplementary Note 1: Distributed Raman amplification in DCF

For this experimental scheme, distributed Raman amplification in the DCF is necessary, because the Rayleigh backscattered light is extremely weak. It can build up enough power through accumulation over a long distance with the help of distributed amplification provided by stimulated Raman scattering (SRS) in the fiber. Raman amplification has an extremely large bandwidth and is capable of amplifying broadband signals. It not only provides gain for incident signal light but also amplifies other oscillating frequency components in the cavity, providing gain for all these frequencies oscillating in the link. However, other wavelengths get amplified due to the extremely large gain bandwidth of Raman amplification, thereby increasing the power of the noise floor. Due to the limited power handling capability of the PD, it is necessary to converge the energy in the loop within the waveband of the signal light emitted by the laser diode so that these broadband frequencies get sufficient power to oscillate in the loop. Therefore, a bandpass optical filter is used between the optical circulator and the EDFA in the loop to suppress power at other wavelengths.

The output power of pump laser is set to 27 dBm in the experiment, making sure that the signal light gets amplified. The pump power is also right below the threshold of a random laser, avoiding the generation of a random laser. This guarantees that the spectral broadening is not caused by the random laser, but by the modulation of the oscillating frequencies.

In the DCF, distributed Raman amplification is used to amplify both forward signal light and the backward Rayleigh scattering[1]. An average power balance model is widely used for describing the features of distributed Raman amplification[2]. It takes into account these important physical effects, including fiber losses, Rayleigh backscattering, Raman gain, and pump depletion. Considering the actual experimental situation, one frequency v_p of Raman pump lightwave and multi-frequencies v_s of modulated lightwave exist in the fiber, and the small spontaneous emission can be neglected. The equations are as follows

$$\frac{dP_p^{\pm}}{dz} = \mp\alpha P_p^{\pm} \mp \sum_{v_s} \frac{g_r\left(\mu, v_s\right)}{K_{eff} A_{eff}} \frac{v_p}{v_s} P_p^{\pm}\left(P_s^{+} + P_s^{-}\right) \quad (1)$$

$$\frac{dP_s^{\pm}\left(v_s\right)}{dz} = \mp\alpha\left(v_s\right) P_s^{\pm} \pm \sum_{v_s} \frac{g_r\left(\mu, v_s\right)}{K_{eff} A_{eff}}\left(P_p^{+} + P_p^{-}\right) P_s^{\pm} + \varepsilon_{v_s} P_s^{\mp} \quad (2)$$

where μ and v_s are optical frequencies of the pump and signal light, respectively. $P_p^{\pm}$ and $P_s^{\pm}$ are the average powers of forward (+) and backward (-) propagating waves along the fiber (z-axis direction), respectively. α is the attenuation coefficient, g_r (μ, v_s) is the Raman gain parameter[3] at frequency v_s by a pump at frequency μ, K_{eff} is a factor related to the polarization state, A_{eff} is the effective cross-sectional area, ε_v is the Rayleigh backscattering coefficient, h is the Planck's constant.

According to experimental parameters (including pump power, optical fiber parameters, etc.) and theoretical calculations, there is an optimal length for the DCF. For a certain signal, it starts to oscillate when its gain is greater than the loss. Therefore, we need to consider its power changes in the loop. In this experimental structure, we should focus on the power changes of the signal light when it enters and exits the DCF. All frequencies within the passband can oscillate, and for each frequency, there are many equally spaced scattering points in the

fiber that meet the phase-matching condition. Therefore, the Rayleigh scattered light power reflected at the input port of the DCF is the sum of the reflected power of all these frequencies at all scattering points, which is a very complicated process. It is difficult to select the appropriate fiber length by calculating the optical power of a certain frequency in the passband. Therefore, we consider a rough calculation of an appropriate fiber length from the perspective of the changes in the gain and loss in the fiber.

Because the backward Rayleigh scattering is extremely weak, even if there is distributed amplification provided by stimulated Raman scattering in the fiber, its power is not large enough to make the signal oscillate in the loop. An additional optical amplifier is needed to amplify the backward Rayleigh scattering light in the loop. Therefore, we hope that as much backward Rayleigh scattered light as possible in the DCF can return to the loop to obtain higher optical power. It can be considered that the length of the optical fiber during which the pump light can amplify the signal light is the optimal length. As the signal light extracts the energy of the pump light and the fiber loss during the pumping process, the power of the pump light will gradually decrease. When the signal light power reaches half of the pump light power, it can be considered that the pump light can no longer amplify the signal light, that is, the pump exhaustion phenomenon occurs. According to the actual experimental parameters given in the article and the power balance model of the Raman amplification process, we have calculated a length of 15 km within which the pump light can continuously provide gain for the signal light. There are many factors that determine the length, such as the effective cross-sectional area of the fiber, the loss of the pump light and signal light in the fiber, and the Raman gain coefficient. The larger the effective cross-sectional area of the optical fiber, the stronger the backward Rayleigh scattering. If the pump light loss is smaller, a longer fiber can be used to obtain greater backscatter power.

Supplementary Note 2: The influence of SBS on signal randomness and the suppression method

If SBS is not completely suppressed, the randomness of the generated signal will be affected. An experiment is performed to compare the system state when there is a strong

SBS and when there is no SBS.

The state of the system can be observed from the phase space. In mathematics and physics, phase space is a space used to represent all possible states of a system; each possible state of the system has a corresponding point in the phase space. For random signals, the phase space shows evenly distribution of scattered points; when the signal deviates from a perfect random oscillation, the system state in the phase space will show a certain trend distribution. For example, the system state distribution in the phase space of a single-frequency signal shows a closed loop trajectory. The phase space of the chaotic signal presents a fractal structure.

As shown in Supplementary Fig. 1, we use an electrical filter with a bandwidth of 8-12 GHz in the loop, and measure the waveform and spectrum in two cases. Case I: No SBS is observed in the spectrum. The signal spectrum is shown in Supplementary Fig. 1a. And the reconstructed phase space displays an evenly scatter plot in Supplementary Fig. 1b. Case II: There is a significant SBS frequency shift at 9.8GHz in the spectrum shown in Supplementary Fig. 1c. It can be seen from the reconstructed phase space in Supplementary Fig. 1d that the system state deviates from the evenly distributed scatter pattern, showing a certain trend. When the power of the SBS frequency shift component is higher, the system state deviation is more obvious. Consider an extreme situation, that is, when the SBS frequency shift power is dominant, the phase space will transition from the scattered point distribution to the closed circle distribution. When the noise power is lower, the closed circle is closer to a perfect circle. When the system deviates from a perfect random oscillation, this trend of the phase space will be reflected to the waveform. Even small deviations can be seen from the phase space. The random numbers generated by such a device will also be affected by non-random factors and may not be regarded as a perfect random number.

In order to allow more laser power be injected into the DCF and avoid the influence of SBS, we should take reasonable measures to suppress SBS. Phase modulation on the input laser is a commonly used effective method to suppress SBS. An experiment is

performed with phase modulation. First, we disconnect the feedback electrical signal to the MZM (open loop). The optical spectrum is shown in red in Supplementary Fig. 2a, and a clear SBS frequency shift can be seen. Then, an 8 Gs/s Pseudo-Random Binary Sequence is used to modulate the optical carrier through a phase modulator placed between the laser and the MZM, and the spectrum result is as shown by the blue line. The spectrum of the blue line is obviously broadened, and SBS is suppressed. At the same time, we observed the noise floor obtained from the PD before and after modulation through a spectrum analyzer, as shown in Supplementary Fig. 2b. The open-loop noise floor signal when no phase modulation is applied is shown in the red spectrum. When phase modulation is used, the noise floor is obviously raised from DC to several GHz. Although the spectrum is only slightly broadened, it generates several GHz additional noise signals when mapped in the microwave domain. Generally, when phase modulation is used, the modulated signal cannot be detected from the PD. However, the random distribution feedback in the fiber and the abnormal dispersion of the DCF will change the phase relationship of light, thus realizing the transition from phase modulation to intensity modulation. The PRBS signal through phase modulation is detected from the PD. In order not to confuse this uplift of the noise floor caused by the application of external modulation with its own oscillation signal after the OEO feedback, phase modulation is not adopted in the experiment. We only suppress the SBS process by reducing the signal optical power. However, this method of phase modulation is still very effective. In subsequent experiments, the phase modulation method can be used to increase the power of the optical carrier input to the DCF.

Supplementary Note 3: The oscillation process arising from noise

The oscillation process arising from noise is also recorded, as shown in Supplementary Fig. 3. The waveform is sampled from the broadband random OEO when the radiofrequency filter centered at 5 GHz with a 3-dB bandwidth of 60 MHz is used in the cavity. As can be seen, all frequencies in the passband start oscillating from noise simultaneously. From this process, it can also reflect the randomness of Rayleigh scattering.

Supplementary Note 4: Random signal spectrum with ultra-narrow bandwidth

By replacing the filter with this structure, all frequencies in the passband range can be vibrated, regardless of the filter bandwidth. In the case of using an ultra-narrowband filter, the single-frequency signal will not be filtered out due to the role of the filter. A narrow-band electrical filter with a center frequency of 10 GHz and a 3-dB bandwidth of 29 MHz is used in the loop. The spectrum is shown in Supplementary Fig. 4. It can be seen from the spectrum that all frequencies within the passband of the filter can oscillate in the loop. Therefore, no matter how narrow the filter bandwidth is, all frequencies in its passband can get enough gain and oscillate in the loop. Single-frequency oscillation cannot be achieved simply by changing a narrower filter.

Supplementary Note 5: Distinguishing the random signal from a chaotic signal

The non-periodic and irregular characteristics of a signal generated in the broadband random OEO, which appears unpredictable, has similar characteristics to chaos. Broadband chaos can also be generated in an OEO, where the modulator has a nonlinear transmission function of the applied voltage and works as a nonlinear element. As a result, the signal generated in the chaotic OEO displays plenty of dynamical behaviors.

Here, we constructed a chaotic OEO as a comparison of our broadband random OEO. The chaotic OEO link is similar to that mentioned in ref. [4], shown in Supplementary Fig. 5. The light wave generated by an LD is sent to a Mach-Zehnder modulator. The output from MZM gets amplification in the EDFA after passing through a short fiber, and then converted to an electrical signal in a photodetector (PD). A fraction of the microwave signals is sent to a spectrum analyzer and a real-time oscilloscope for analysis. The other part of signals is amplified through an EA and sent to the MZM to form a closed loop. The output lightwave of LD is amplified by the EDFA to 13 dBm and then received by the PD. Other parameters are the same as in the broadband random OEO in our experiment. As the optical power entering the PD increasing, the system gradually enters the chaotic state. The waveform of the chaotic signal is sampled with a real-time oscilloscope at a sampling rate of 100 GS/s, as shown in Supplementary Fig. 6a. The waveform is chaotic and irregular. The attractor in phase space

shown in Supplementary Fig. 6b is derived from the time series of chaotic OEO, which shows fractal orbits.

Supplementary Note 6: The application on secure communications of the broadband random OEO

In addition to the application in the noise radar mentioned in the text, the wideband random OEO can also be used in secure communications for the encryption of the information, although how to decrypt the information by the intended receiver without the risk of being eavesdropped still requires further investigation. The random signal generated by the proposed OEO can be used as a key to encrypt information. The device can be placed at a distance between the two communicating parties, and the same random signal can be obtained by precisely controlling the delay between the two parties to the device. The sender uses the random signal to hide the information to encrypt it and deliver it to the receiver. After the receiver receives the signal, it can decrypt and obtain the information by strictly synchronizing the delay and power of the encrypted signal and the random signal, and performing differential processing on the two. It might be difficult for eavesdroppers to accurately control the time delay, so the random signal obtained cannot be differentially processed with the encrypted information to decrypt the information. This method may be used for secure communication, but it also has the risk of eavesdropping. We hope to find a safer way to prevent eavesdropping in the follow-up work.

Supplementary References

1. Bromage, J. Raman amplification for fiber communications systems. *J. Lightwave Technol.* **22**, 79 (2004).
2. Min, B., Lee, W. J., & Park, N. Efficient formulation of Raman amplifier propagation equations with average power analysis. *IEEE Photonics Technol. Lett.* **12**, 1486-1488 (2000).
3. Stolen, R. H., Ippen, E. P., & Tynes, A. R. Raman oscillation in glass optical waveguide. *Appl. Phys. Lett.* **20**, 62-64 (1972).
4. Callan, K. E., Illing, L., Gao, Z., Gauthier, D. J., & Schöll, E. Broadband chaos generated by an optoelectronic oscillator. *Phys. Rev. Lett.* **104**, 113901 (2010).

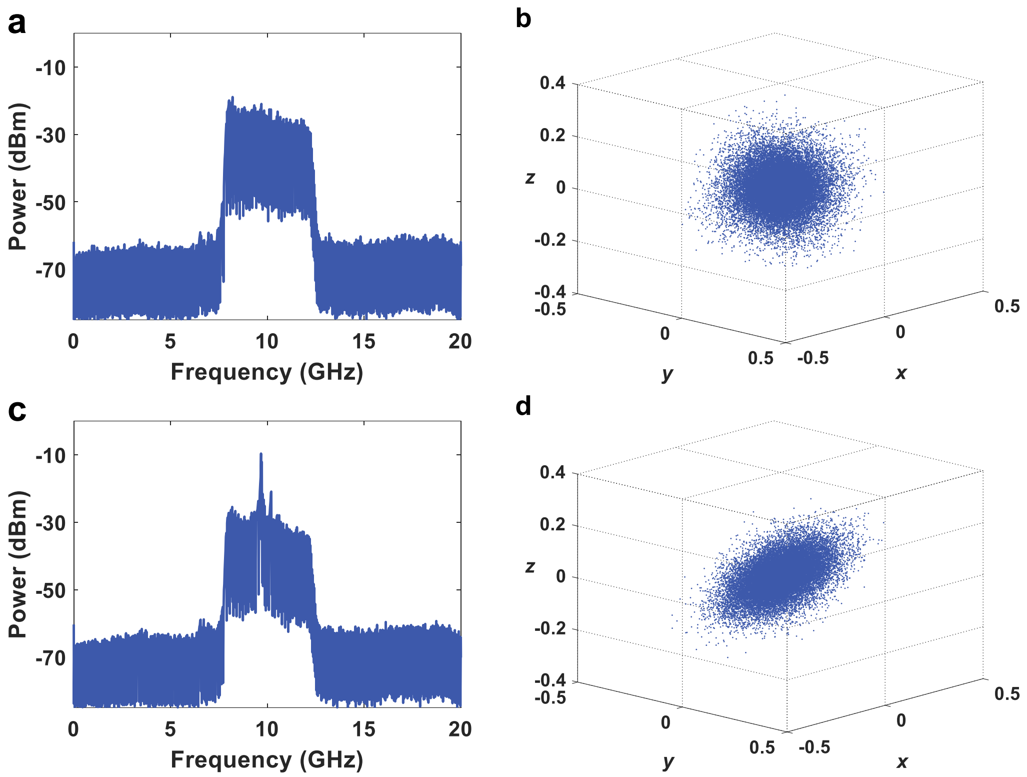

Supplementary Fig. 1 Spectrum(a) and phase space (b) results when SBS is not present. Spectrum (c) and phase space (d) results in the presence of SBS.

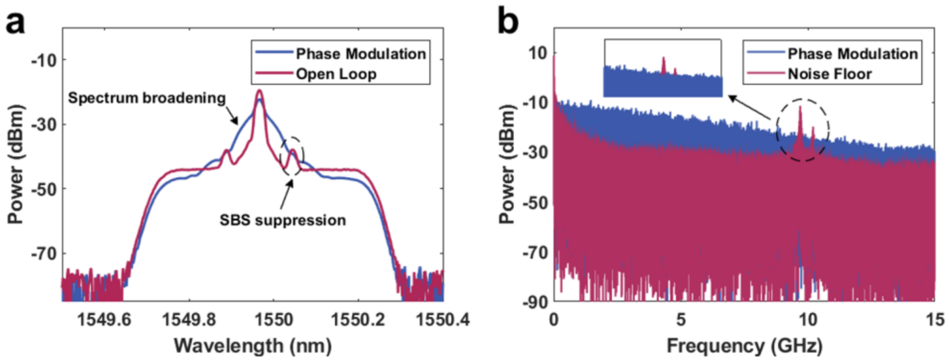

Supplementary Fig. 2 Experiment results with phase modulation. (a) the spectral broadening (blue) with phase modulation on the optical carrier to suppress the SBS. The red line shows spectrum of open loop without modulation. **(b)** Noise floor when phase modulation broadens the spectrum (blue) and when there is no modulation(red).

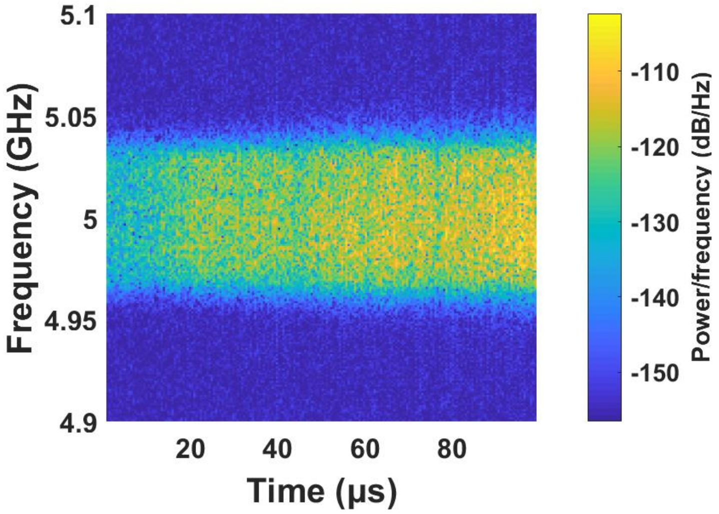

Supplementary Fig. 3 Real-time frequency distribution of the temporal waveform when the OEO starts to oscillate.

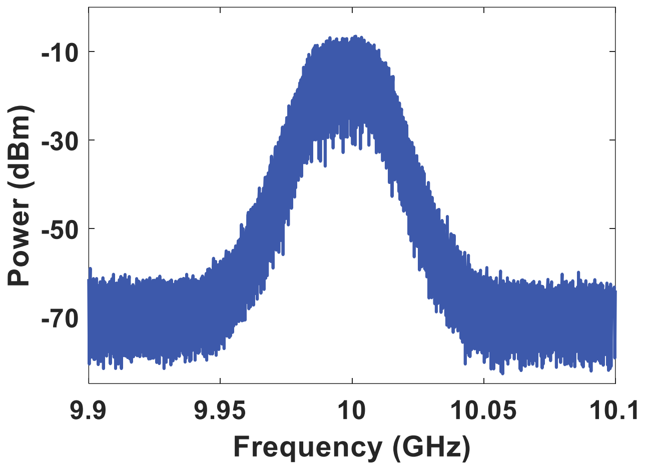

Supplementary Fig. 4 Spectrum results when an ultra-narrow filter is used in the OEO. The narrow-band electrical filter is centered at 10 GHz with a 3-dB bandwidth of 29 MHz. All frequencies within the passband of the filter can oscillate in the loop no matter how narrow the bandwidth of the filter is.

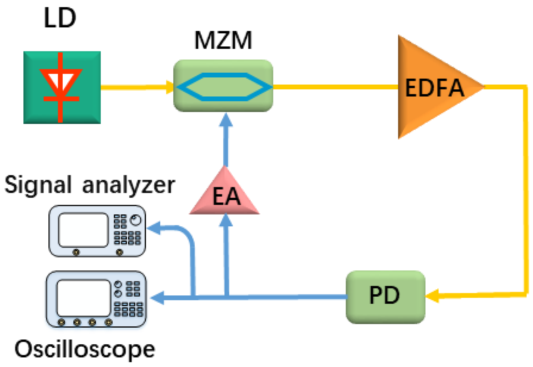

Supplementary Fig. 5 Block diagram of the chaotic OEO. LD, laser diode; MZM, Mach-Zehnder modulator; EDFA, erbium-doped fiber amplifier; PD, photodetector; EA, electrical amplifier.

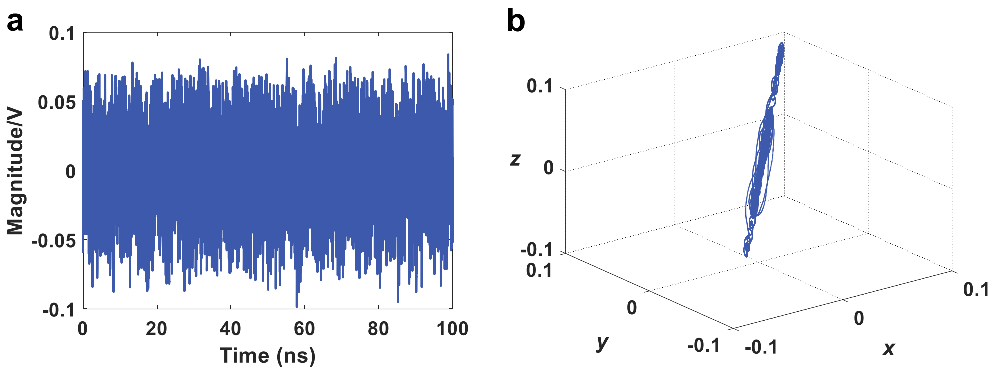

Supplementary Fig. 6 Experiment results of a signal generated in the chaotic OEO. (a) Temporal waveform of the chaotic signal. The waveform is chaotic and irregular. **(b)** The phase space and a broadband random OEO. The reconstructed attractor in the phase space from a time series sampling from a signal generated in the chaotic OEO, showing fractal orbits.
